# Supplementary material for: Development of Salt- and Gastric-Resistant Whey Protein Isolate Stabilized Emulsions in the Presence of Cinnamaldehyde and Application in Salad Dressing
Source: Foods. 2021 Aug 12;10(8):1868. doi: 10.3390/foods10081868 (PMC8394555; doi:10.3390/foods10081868)
Supplement: Supplementary file 1 [file foods-10-01868-s001.zip › foods-1310171-supplementary.pdf]

Supplementary material

Figure S1. Effect of ion type and concentration on the zeta-potential of WPI solutions.

Figure S2. Appearance of M100C0 and M70C30 emulsions during digestion in the presence of different ions.

Figure S1

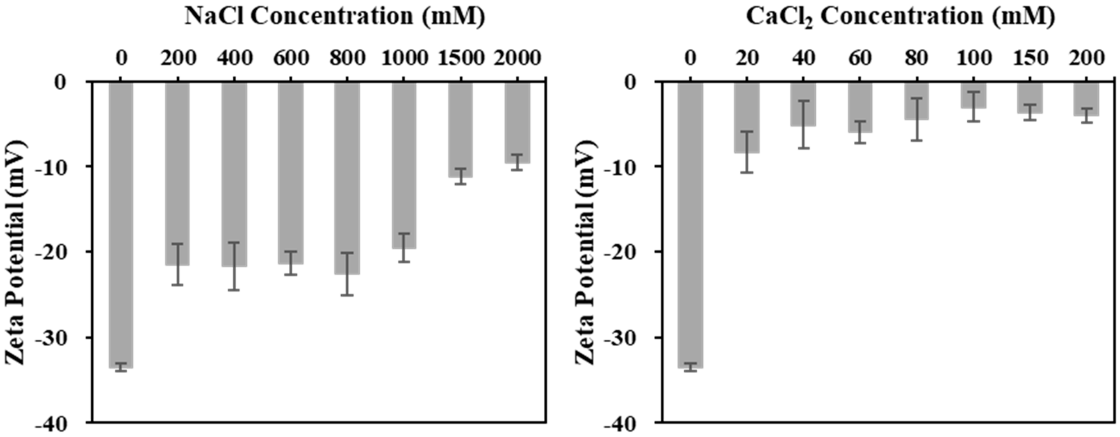
NaCl Concentration (mM)
0
200
400
600
800
1000
1500
2000
Zeta Potential (mV)
0
-10
-20
-30
-40
CaCl2 Concentration (mM)
0
20
40
60
80
100
150
200
Zeta Potential (mV)
0
-10
-20
-30
-40

Figure S2

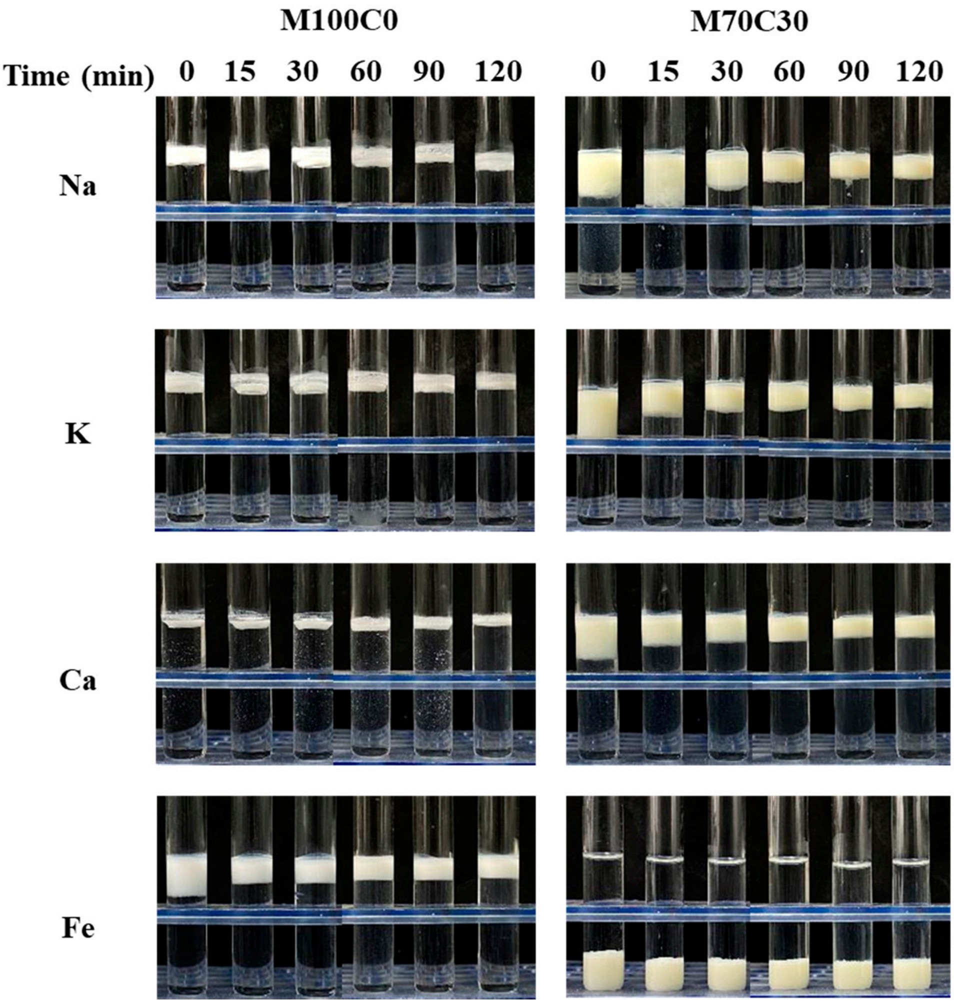
M100C0
M70C30
Time (min)
0
15
30
60
90
120
0
15
30
60
90
120
Na
K
Ca
Fe
